# Blast Exposure, White Matter Integrity, and Cognitive Function in Iraq and Afghanistan Combat Veterans

**DOI:** 10.3389/fneur.2017.00127

**Published:** 2017-04-21

**Authors:** Iliyan Ivanov, Corey Fernandez, Effie M. Mitsis, Dara L. Dickstein, Edmund Wong, Cheuk Y. Tang, Jessie Simantov, Charlene Bang, Erin Moshier, Mary Sano, Gregory A. Elder, Erin A. Hazlett

**Affiliations:** ^1^Department of Psychiatry, Icahn School of Medicine at Mount Sinai, New York, NY, USA; ^2^Research & Development, James J. Peters Veterans Affairs Medical Center, Bronx, NY, USA; ^3^Rehabilitation Medicine Service, James J. Peters Veterans Affairs Medical Center, Bronx, NY, USA; ^4^Department of Pathology, Uniformed Service University of Health Science, Bethesda, MD, USA; ^5^Fishberg Department of Neuroscience, Friedman Brain Institute, Icahn School of Medicine at Mount Sinai, New York, NY, USA; ^6^Department of Radiology, Icahn School of Medicine at Mount Sinai, Translational and Molecular Imaging Institute, New York, NY, USA; ^7^Department of Rehabilitation Medicine, Icahn School of Medicine at Mount Sinai, New York, NY, USA; ^8^Department of Population Health Science and Policy, Icahn School of Medicine at Mount Sinai, New York, NY, USA; ^9^Department of Neurology, Icahn School of Medicine at Mount Sinai, New York, NY, USA; ^10^Neurology Service, James J. Peters Veterans Affairs Medical Center, Bronx, NY, USA; ^11^Mental Illness Research, Education, and Clinical Center (MIRECC VISN 2 South), James J. Peters Veterans Affairs Medical Center, Bronx, NY, USA

**Keywords:** adult brain injury, diffusion tensor imaging, magnetic resonance imaging, cognitive function, military injury

## Abstract

The long-term effects of blast exposure are a major health concern for combat veterans returning from the recent conflicts in Iraq and Afghanistan. We used an optimized diffusion tensor imaging tractography algorithm to assess white matter (WM) fractional anisotropy (FA) in blast-exposed Iraq and Afghanistan veterans (*n* = 40) scanned on average 3.7 years after deployment/trauma exposure. Veterans diagnosed with a blast-related mild traumatic brain injury (mTBI) were compared to combat veterans with blast exposure but no TBI diagnosis. Blast exposure was associated with decreased FA in several WM tracts. However, total blast exposure did not correlate well with neuropsychological testing performance and there were no differences in FA based on mTBI diagnosis. Yet, veterans with mTBI performed worse on every neurocognitive test administered. Multiple linear regression across all blast-exposed veterans using a six-factor prediction model indicated that the amount of blast exposure accounted for 11–15% of the variability in composite FA scores such that as blast exposure increased, FA decreased. Education accounted for 10% of the variability in composite FA scores and 25–32% of FA variability in the right cingulum, such that as level of education increased, FA increased. Total blast exposure, age, and education were significant predictors of FA in the left cingulum. We did not find any effect of post-traumatic stress disorder on cognition or composite FA. In summary, our findings suggest that greater total blast exposure is a contributing factor to poor WM integrity. While FA was not associated with neurocognitive performance, we hypothesize that FA changes in the cingulum in veterans with multiple combat exposures and no head trauma prior to deployment may represent a marker of vulnerability for future deficits. Future work needs to examine this longitudinally.

## Introduction

The long-term effects of blast exposure have become a growing health concern within the military due to the large number of Iraq and Afghanistan combat veterans who have experienced single or multiple blasts from improvised explosive devices, rocket propelled grenades, and mortar rounds. It is estimated that approximately 10–20% of veterans returning from these conflicts sustained blast-related mild traumatic brain injury (mTBI) in theater (1–4). The true prevalence may be even higher, given that many blast-related injuries went unrecognized both during and after deployment, particularly in the early years of these conflicts (5). Screening and diagnosing of mTBI are complicated by the nature of the condition as initial assessment heavily relies on self-report measures (2, 6, 7), and the overlap of TBI symptoms with those of post-traumatic stress disorder (PTSD), which is present in at least 30% of returning service members who have suffered a mTBI (1, 2, 4).

### Blast Exposure and Cognitive Functioning

The chronic effects of blast exposure on cognitive functioning are an area of growing research interest. One study found that Marines returning from combat with self-reported concussion and symptoms consistent with mTBI showed decline of cognitive performance on a computerized cognitive battery at a 3-month follow-up; however, these deficits recovered by month 6 (8). By contrast, another group reported that veterans with blast-related mTBI and combat controls with no history of blast exposure showed no performance differences on cognitive measures including the Controlled Oral Word Association Test, Trail Making Test, Color–Word Interference Test, and Verbal Selective Reminding Test (9). The interpretation of such discrepancies is further complicated by the use of different methodologies to assess cognition (i.e., raw scores from paper and pencil tests vs. reaction time on computerized tests). A review paper by Karr et al. (10) points to inconsistencies in the conceptualization of cognitive constructs used to describe post-mTBI impairment. Moreover, the authors stipulate that the magnitude of mTBI-related effects within each cognitive domain remains unclear as the respective effect sizes for “cognitive deficits” appear especially heterogeneous across meta-analyses (i.e., *d* = −0.11 to 0.72) and that these higher order functions appear most susceptible to multiple mTBI [*d* = 0.24 (11)]. Recent reports also suggest that mTBI patients may, in fact, experience subtle cognitive deficits that reflect diminished initial acquisition of novel information (12, 13). Specifically, mTBI patients demonstrated statistically significant deficits in performance on the first trial of the California Verbal Learning Task, whereas there were no impairments on total learning or memory composite variables (12). The most relevant hypothesis is that mTBI patients may demonstrate cognitive deficits that are not often detected in standard neuropsychological assessments. In short, the nature and extent of cognitive impairment following blast exposure are varied in the literature, symptomatic complaints are common, and they do not seem to associate exclusively with any single profile.

### Diffusion Imaging and Blast mTBI

More recently, neuroimaging techniques have been utilized to help elucidate the pathology of mTBI. While there have been several neuroimaging studies devoted to civilian TBI (14–17), the extent to which blast injuries are a distinct neurobiological entity remains unknown but has become the focus of much recent work [reviewed in Ref. (18)]. Diffusion tensor imaging (DTI), a magnetic resonance imaging (MRI) method, assesses the diffusion of water molecules in order to map microscopic details about the integrity of white matter (WM) fiber structure. It is based on the principle that water moves most easily along axonal bundles because there is the smallest number of obstacles to prevent movement in this direction. Diffusion measurements along different axes are fitted to a 3D ellipsoid (tensor) at each voxel. The most common metric in DTI studies is fractional anisotropy (FA), which reflects the magnitude and directionality of water diffusion. More specifically, FA represents the fraction of the tensor that can be assigned to diffusion that is constrained along one axis only, referred to as anisotropic diffusion. FA values are scaled from 0 (isotropic) to 1 (anisotropic). FA and other DTI metrics are thought to reflect the microstructural properties of WM tracts, including abnormal axonal diameter, coherence of the fiber tracts, fiber density, and myelination, although the microstructural correlates are not entirely clear (19–23). Because DTI is considered to be sensitive to subtle forms of damage and to diffuse axonal injury, it holds promise as a sensitive tool for identifying subtle pathology in blast-related mTBI, and several studies have utilized DTI to examine blast injury across a range of TBI severity at various time intervals post-exposure (24–34). These studies have generally found decreased FA in blast-related mTBI, results that have been interpreted as suggesting that diffuse axonal injury is a major component of the structural injury associated with blast. Nevertheless, the relationship between the total amount of blast exposure, such as the number of blasts, and the development of clinical symptoms of mTBI remains unclear. More specifically, (a) there are no studies that have specifically looked at the relationship between total number of blasts, especially blasts not linked to any physical or psychological sequela; and (b) there is little consistent evidence linking blast exposure with persistent changes in brain morphology and physiology.

The current study used an optimized DTI tractography algorithm to measure FA and to characterize the effects of blast exposure on WM integrity in blast-exposed Iraq and Afghanistan veterans, who had no formally diagnosed history of head trauma prior to deployment. Loss of consciousness (LOC) and co-occurring PTSD were investigated as indicators of blast severity, and veterans who suffered a blast-related mTBI were compared to a group of combat veterans who had a history of blast exposure but did not meet criteria for a TBI diagnosis. Finally, we examined the relationship between total amount of blast exposure, WM integrity, and cognitive performance, hypothesizing that veterans exposed to more blasts would have lower FA. We also predicted that lower FA, indicative of compromised WM integrity, would be associated with poorer performance on neuropsychological tests across cognitive domains.

## Methods

### Participants

Forty Iraq and/or Afghanistan male veterans were recruited through the James J. Peters Veterans Affairs Medical Center (JJPVAMC, Bronx, NY, USA) Polytrauma/TBI Clinic through referrals from study physicians (Jessie Simantov and Gregory A. Elder), neuropsychologists (Effie M. Mitsis and Charlene Bang), as well as through flyers and advertisements posted throughout the medical center. Participants had an average age of 32.8 ± 7.6 years (range: 22–50 years) and 14.2 ± 1.9 years of education (Table 1). Participants were enrolled 3.7 ± 1.7 years post-deployment (range: 0.2–7.0 years). All participants had a history of primary blast exposure, with 24 veterans experiencing blasts severe enough to meet criteria for mTBI (Tables 2 and 3). The number of blast events experienced by study participants varied widely (range: 1–45, mean/SD 8.2 ± 9.8). Participants who met criteria for blast-related mTBI experienced an average of 4.8 ± 5.0 blast episodes during the course of their deployment, while veterans with no TBI diagnosis experienced more blasts despite not meeting threshold for a TBI diagnosis (mean/SD 13.1 ± 13.0; *t* = 2.85, df = 38, *p* < 0.01). None of the veterans studied had a formally diagnosed history of head injury prior to military service.

**Table 1:** **Sample characteristics**.

Variable	*n*	Mean ± SD
Age	40	32.75 ± 7.61
Education (years)	40	14.16 ± 1.92
Post-deployment (years)	40	3.66 ± 1.73
Total # of blasts	40	8.15 ± 9.81
PCL-M score	33	44.12 ± 17.58
PTSD (% meeting PTSD criteria)	33	42.42%
Handedness (% right handed)	40	92.50%

*PCL-M, Post-Traumatic Stress Disorder Checklist-Military Version; PTSD, post-traumatic stress disorder*.

**Table 2:** **Type of blast exposure**.

	Primary	Secondary	Tertiary	Quaternary
All study participants (*n* = 40)	40	17	16	7
Participants without blast mild traumatic brain injury (mTBI) (*n* = 16)	16	1	3	0
Participants with blast mTBI (*n* = 24)	24	15	12	7

*Primary exposure defined as direct effect of blast overpressure on tissue*.

*Secondary exposure defined as injury from objects propelled by the blast*.

*Tertiary exposure defined as injuries resulting from an individual being propelled through the air and striking other objects*.

*Quaternary exposure defined as all other injuries caused by explosions, such as burns, crush injuries, and toxic inhalations*.

**Table 3:** **Blast exposure subgroups**.

	Primary only	Primary and secondary	Primary and tertiary	Primary and quaternary	Primary, secondary, and tertiary	Primary, secondary, tertiary, and quaternary
No blast mild traumatic brain injury (mTBI) (*n* = 16)	12	1	3	0	0	0
Blast mTBI (*n* = 24)	6	5	2	1	4	6

*Shows the combinations of different types of blast exposure for the participants in the study*.

The PTSD Checklist-Military Version (PCL-M) was administered to evaluate PTSD symptomatology. We determined the presence of PTSD using a cutoff score of 44, in accordance with the guidelines published by the National Center for PTSD.^1^ Fourteen of 33 veterans (42%) who completed the PCL-M met criteria for PTSD (note: 7 participants did not complete the measure). PCL-M scores were significantly higher in veterans with blast-related mTBI (mean/SD 50.9 ± 16.7) than those without (mean/SD 36.9 ± 16.0; *t* = −2.45, df = 31, *p* = 0.02).

Exclusion criteria included any significant medical illness, neurological disease or psychiatric disorder (other than depression and/or PTSD), moderate-to-severe TBI, systemic cancer, history of psychoactive substance use/abuse and current alcohol or other drug dependence within the past year, use of psychoactive drugs, any significant lifetime history of pre-combat-related TBI with LOC or reported history of concussion requiring hospitalization, education level <10 years, or the presence of any MRI-incompatible prostheses or ferromagnetic metal. All participants had a negative urine toxicology screening for drugs of abuse on the day of their MRI scan. All study procedures were approved by the JJPVAMC and Icahn School of Medicine at Mount Sinai Institutional Review Boards. Participants provided written informed consent and were paid for their time and travel.

### Clinical Assessments

Details of blast injury were determined by self-report and review of all available clinical histories from the Veteran’s Affairs (VA) medical records of each participant (Tables 2 and 3). Primary blast injury refers to the direct effect of blast overpressure on tissue. Secondary injury results from objects propelled by the blast. Tertiary injuries are a feature of high-energy explosions and occur as a result of an individual being propelled through the air and striking other objects. Quaternary blast injuries encompass all other injuries caused by explosions, such as burns, crush injuries, and toxic inhalations. Assessments for the type of blast injury followed the accepted standards (35–37); all participants had a negative structural MRI scan indicating no brain injuries secondary to impact from foreign objects.

Prior to entry in the study, all veterans were evaluated by study physicians as part of their standard TBI screening and medical evaluation and underwent neuropsychological evaluation. A final diagnosis of blast-related mTBI was based upon Department of Defense/VA criteria (38) and determined by consensus during weekly meetings of the clinical and TBI/Polytrauma team. Study personnel obtained additional detailed history of blast exposure through interview with each veteran. The Defense and Veterans Brain Injury Center screening tool was administered to obtain information regarding any transient loss of awareness or LOC (≤30 min), post-traumatic amnesia (<24 h), as well as information on post-concussive symptoms of dizziness, confusion, headache, balance and memory problems, tinnitus, irritability, and sleep problems.

### Neurocognitive Assessments; Domain and Composite Scores

Participants completed a battery of neuropsychological tests assessing cognitive domains with a particular emphasis on memory, executive function, attention, and language. These included the Wechsler Adult Intelligence Scale-Fourth Edition (39), which assesses participants’ overall intelligence; Wide Range Achievement Test 4 (40), which measures an individual’s ability to read words, comprehend sentences, spell, and compute solutions to math problems; Controlled Oral Word Association Test-FAS [COWA-FAS (41)], which is a verbal fluency test measuring spontaneous production of words belonging to the same category or beginning with some designated letter; California Verbal Learning Test (CVLT)-Second Edition (42, 43), which measures episodic verbal learning and memory and can detect sensitivity to a range of clinical conditions; and the Brief Visuospatial Memory Test-Revised (44), which measures figural learning and retention for examination of non-verbal memory (Table 4). The instruments were administered and scored by trained research assistants, and the final scoring was confirmed by a neuropsychologist. Raw scores for each test were standardized based on age- and education-adjusted normative data. Tests were grouped into three domains: attention/executive function, language/education, and memory. Domain scores were calculated by averaging the normalized test score within each cognitive domain. An overall composite score was calculated by averaging the three domain scores. This approach to calculating domain and composite scores has been used by our group and others (45, 46) in studies evaluating cognitive functioning in mildly-impaired cohorts.

**Table 4:** **Neuropsychological testing performance**.

	Descriptive statistics		Blast subjects vs. normal performance (%)		Correlation with total blast exposure	Blast TBI vs. non-TBI	
	*n*	Mean ± SD	1.5 *z*-scores below mean	2 *z*-scores below mean	*r*	*t*	*p*
***Overall composite score***	32	0.64 ± 0.10			0.31	2.26	**0.032**
**Attention/executive function domain**	33	0.62 ± 0.07			0.01	1.67	0.105
WAIS-IV							
Digit symbol/coding	39	64.26 ± 15.94	3 (7.7)	1 (2.6)	−0.16	0.63	0.535
Symbol search	36	30.78 ± 6.78			−0.72	0.03	0.979
Block design	34	45.71 ± 10.93	0 (0.0)	0 (0.0)	−0.19	0.61	0.544
Digit span forwards	32	10.41 ± 2.15			0.13	3.33	**0.002**
Digit span backwards	32	7.94 ± 2.11			−0.04	1.95	0.059
Letter–number sequencing	36	18.39 ± 4.79			0.23	2.20	**0.035**
Stroop Color–Word Interference	37	42.22 ± 11.71			**0.58**	2.06	**0.047**
Trail Making Test Part A (s)	39	30.72 ± 10.90	2 (5.1)	2 (5.1)	0.06	0.16	0.872
Trail Making Test Part B (s)	39	76.41 ± 31.13			−0.02	1.24	0.223
**Language/education domain**	39	0.69 ± 0.11			0.23	2.96	**0.006**
WRAT4 Word List Reading (standard)	39	102.77 ± 11.45			0.13	2.03	**0.040**
WAIS-IV similarities	39	25.49 ± 5.46			0.22	3.29	**0.002**
COWA-FAS	39	39.59 ± 12.52	6 (15.4)	3 (7.7)	0.20	2.11	**0.042**
**Memory domain**	36	0.59 ± 0.15			0.29	1.46	0.156
CVLT-II total trials 1–5	39	45.54 ± 9.04	5 (12.8)	3 (7.7)	−0.05	1.17	0.249
CVLT-II long delay free recall	39	8.62 ± 3.49	13 (33.3)	3 (7.7)	0.19	0.20	0.844
BVMT-R delayed recall	39	8.69 ± 3.11			0.21	2.19	**0.035**
Rey complex figure delayed recall	37	18.28 ± 6.43			0.32	1.27	0.211

*WAIS-IV, Wechsler Adult Intelligence Scale-Fourth Edition; WRAT4, Wide Range Achievement Test 4; COWA-FAS, Controlled Oral Word Association Test-FAS; CVLT, California Verbal Learning Test-Second Edition; BVMT-R, Brief Visuospatial Memory Test-Revised*.

*Values in bold font are significant at p < 0.05 level*.

### Magnetic Resonance Imaging

Magnetic resonance imaging scans were conducted in the Department of Radiology at the Mount Sinai Hospital, the academic-affiliate hospital of the JJPVAMC. All MRI scans were visually inspected by a neuroradiologist at Mount Sinai blind to diagnostic group. No visible lesions were detected for any veteran, and all research MRI reports were read as normal.

All participants underwent MRI on a Siemens Allegra 3 T head-dedicated scanner for acquisition of axial structural and diffusion tensor images. A pulsed-gradient spin-echo sequence with echo planar imaging pulse sequence acquisition was used. A *b*-factor of 1,250 s/mm^2^ was chosen based on tests performed to find the optimal balance for signal-to-noise ratio and diffusion weighting. Twelve gradient directions with *b* = 1,250 s/mm^2^ were used [repetition time (TR) = 4,100 ms, echo time (TE) = 80 ms, field of view (FOV) = 21 cm, matrix 128 × 128, 28 slices, thickness = 3 mm, skip = 1 mm]. High-resolution 3D MP-RAGE images were also obtained (TR = 2,500 ms, TE = 4.4 ms, FOV = 21 cm, matrix size = 256 × 256, 208 slices with thickness = 0.82 mm). Quality assurance was performed immediately after each scan.

### DTI Processing and Fiber Tracking

FSL^2^ was used for eddy current correction, brain extraction, and the computation of FA and mean diffusivity (MD) maps. DTI tractography processing was performed using in-house software, developed in MATLAB v2013 (The MathWorks Inc., Natick, MA, USA). A multiple region brute-force fiber tracking method was used for quantification of white mater characteristics as previously described (47). Briefly, fibers were traced using a streamline tractography algorithm from every voxel throughout the entire volume that exceeded a minimum FA (48). Tracking was terminated when FA fell below 0.1 or when the algorithm encountered a sharp angle change in the principal diffusion direction between sequential voxels (45°). Each tract was then indexed such that a queried voxel returns all streamlines that pass through it. All tracts that passed through a target voxel are then associated with that voxel. This method is more robust than streamline tractography alone, in which a single fiber emerges from a voxel.

### Image Analysis

**Figure 1:**
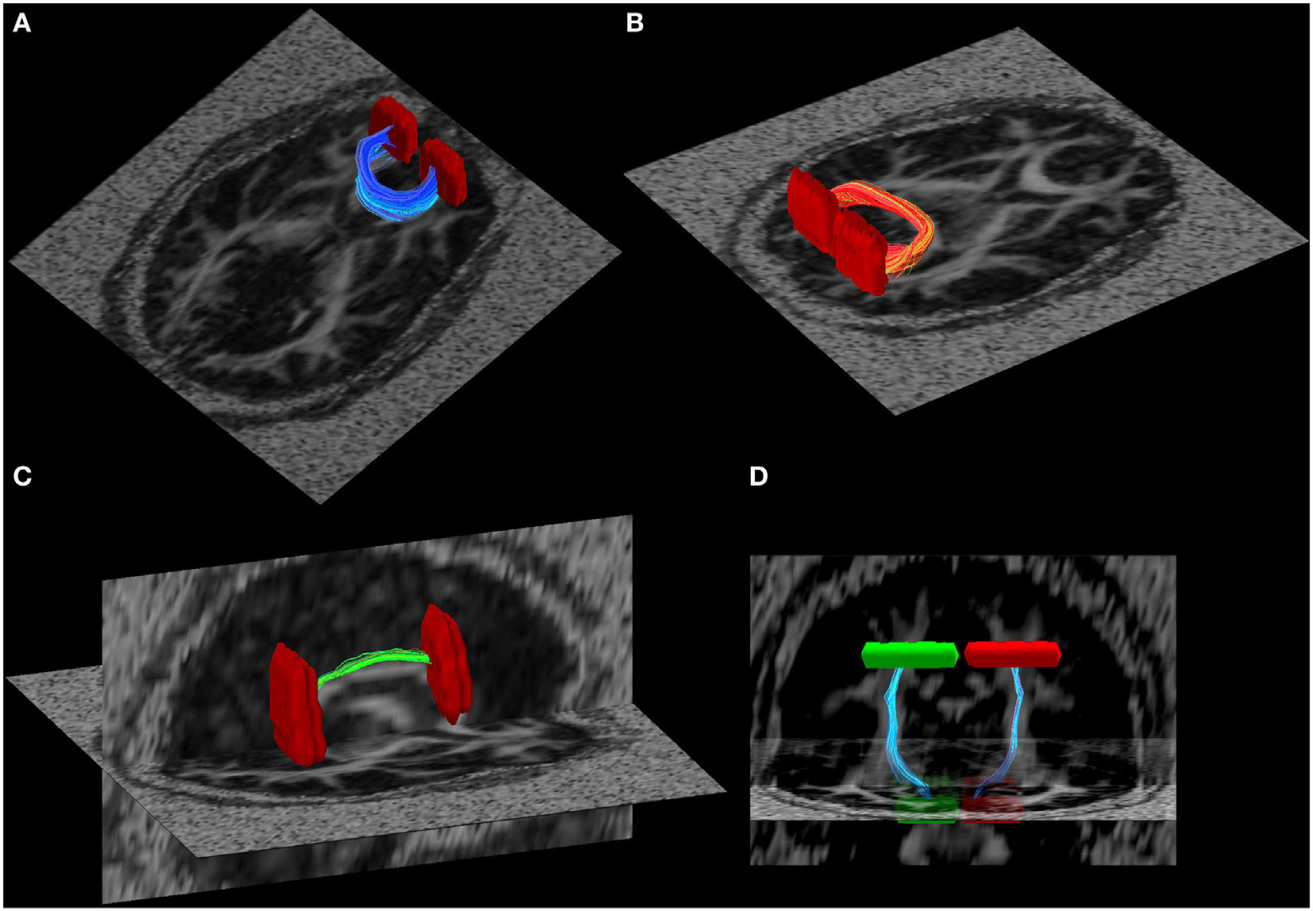
**Illustration of some of the white matter tracts used in the tract-based fractional anisotropy quantification**. **(A)** Forceps major, **(B)** forceps minor, **(C)** R-cingulum, and **(D)** cortical spinal tract. Seed regions of interest are illustrated in red and green.

To minimize variance due to inter-subject differences in tractography seeds, all anatomical criteria were defined on the study-specific template (i.e., mean FA image of all participants in the current study) that was created with the tract-based spatial statistics (TBSS) package of FSL [as in our prior work; see Ref. (49)]. Established methods to create anatomical seed criteria (50) were used to guide diffusion tractography quantification of the following tracts: right and left cingulum bundle, inferior longitudinal fasciculus, superior longitudinal fasciculus, internal capsule, inferior fronto-occipital fasciculus, uncinate, the frontal projection of the corpus callosum (the forceps minor), and the posterior projection of the corpus callosum (the forceps major) (Figure 1). Tract integrity was quantified using a normalized line integral of FA and MD for every tract per participant (49). These results were then exported for further statistical analysis.

In addition, we performed region-of-interest-based FA analysis. Co-registered FA images in Montreal Neurological Institute (MNI) brain space were computed using the TBSS workflow. The Johns Hopkins University ICBM-DTI-81 WM labels atlas was used to locate anatomical regions in MNI152 space. Mean FA was calculated for each atlas-defined region on the each participant’s co-registered MNI FA image computed from TBSS. Composite FA scores were also calculated for each participant (see Statistical Analysis).

### Statistical Analysis

As indicated above, the main objective of this study was to examine the relationship between total amount of blast exposure, WM integrity, and cognitive performance. In order to test our hypotheses that veterans exposed to more blasts would have lower FA and that lower FA would be associated with poorer neurocognitive performance, we employed multiple linear regression models. The variables included in our models were selected based on the consensus that they were clinically important factors that influence WM integrity.

A plot of the residuals vs. predicted values was used to check the assumptions of linearity and homoscedasticity. The normality assumption was evaluated based on the residuals using a QQ plot by comparing the residuals to “ideal” normal observations. A histogram of the Cook’s distance (which is a measure of the influence of each observation on the regression coefficients) was generated to identify outliers that may or may not be influential data points. Multicollinearity was assessed by examining tolerance and variance inflation factors of each variable in the regression models.

Due to the lack of consistent reports indicating relations between altered FA in specific brain regions and specific cognitive deficits, we adopted a two-set analytical approach: (1) to examine the correlations of composite FA across all the key tracts and (2) to examine the FA values of the left and right cingulum bundle-specific cognitive domains. Therefore, we developed two sets of linear regressions. In the first set, separate models were developed with a composite value of FA as the dependent variable. This composite FA value was calculated by averaging the FA values from each participant’s WM tracts (middle cerebellar peduncle, pontine cross tract, genu of corpus callosum, body of corpus callosum, splenium, fornix, corticospinal tract, medial lemniscus, inferior cerebellar peduncle, superior cerebellar peduncle, anterior limb of the internal capsule, posterior limb of internal capsule, retrolenticular internal capsule, anterior corona radiata, superior corona radiata, posterior corona radiata, posterior thalamic radiation, sagittal stratum, external capsule, cingulum bundle, fornix striata terminalis, superior longitudinal fasciculus, superior frontal occipital fasciculus, uncinate fasciculus, and tapetum). PTSD symptom severity (PCL-M score), total blast exposure, LOC, and the four cognitive scores (memory domain score, language/education domain score, attention/executive function domain score, and overall composite score) were the primary independent variables in this set, adjusting for age and education. In the second set, separate models were estimated with the FA values of the left and right cingulum bundle as dependent variables. PTSD symptom severity (PCL-M score), total blast exposure, LOC, and the four cognitive scores (included singly for each WM tract) were the primary independent variables while adjusting for age and education. This analysis was replicated in several other WM tracts implicated in studies of blast injury utilizing diffusion imaging techniques.

A final set of regression models examined neurocognition. Separate models were developed with the domain and composite cognitive scores as dependent variables. PTSD symptom severity (PCL-M score), total blast exposure, LOC, and the composite value of FA were the primary independent variables while adjusting for age and education.

In all models, linear and quadratic terms were explored for the explanatory variables. Semi-partial *R*^2^ estimates were calculated for each of the explanatory variables in each model, and an overall *R*^2^ was calculated for each model. All models were estimated using SPSS statistical software.

Pearson product-moment correlations were used to investigate the association between total blast exposure and cognitive functioning across the entire blast-exposed population. Two-tailed, unpaired *t*-tests examined differences in neuropsychological testing performance, PCL-M scores, and blast exposure between blast-exposed veterans who did and did not meet criteria for mTBI. An ANCOVA (Shapiro–Wilks; covariates were age and education) was used to examine between-group differences in FA: Group (no mTBI vs. mTBI) × WM tract (middle cerebellar peduncle, pontine cross tract, genu of corpus callosum, body of corpus callosum, splenium, fornix, corticospinal tract, medial lemniscus, inferior cerebellar peduncle, superior cerebellar peduncle, anterior limb of the internal capsule, posterior limb of internal capsule, retrolenticular internal capsule, anterior corona radiata, superior corona radiata, posterior corona radiata, posterior thalamic radiation, sagittal stratum, external capsule, cingulate, gyrus, fornix striata terminalis, superior longitudinal fasciculus, superior frontal occipital fasciculus, uncinate fasciculus, and tapetum).

## Results

### Neurocognitive Performance

We first conducted a normative assessment comparing participant scores on certain individual neuropsychological tests to normalized datasets. The tests chosen for this analysis have well-established norms and are highly indicative of the domains outlined in Table 4. The frequency counts (i.e., number of veterans who performed below 1.5 SD below the mean and below 2 SD below the mean for these tests) are listed in the third column of Table 4. On tests of attention and executive function, participants performed close to the mean for their age. For example, not one participant (in either group) performed 1.5 SDs below the mean on block design. On language tests (COWA-FAS), a greater number of participants performed poorly. However, the worst performance was on memory tasks: one-third of participants performed 1.5 SDs or more below the mean for their age on the CVLT.

Next, we examined associations between total blast exposure and cognitive performance. Total blast exposure did not correlate well with performance on neuropsychological tests. Only the Stroop Task was significant: greater number of blasts was associated with poorer performance (all *r* values are reported in Table 4). Finally, on an exploratory basis, we examined whether there were differences between blast-exposed veterans who met criteria for blast-related TBI with those who did not meet criteria. Veterans with mTBI performed worse than the non-TBI veterans on every test administered. Table 4 reports *t* and *p* values.

### WM Integrity

**Figure 2:**
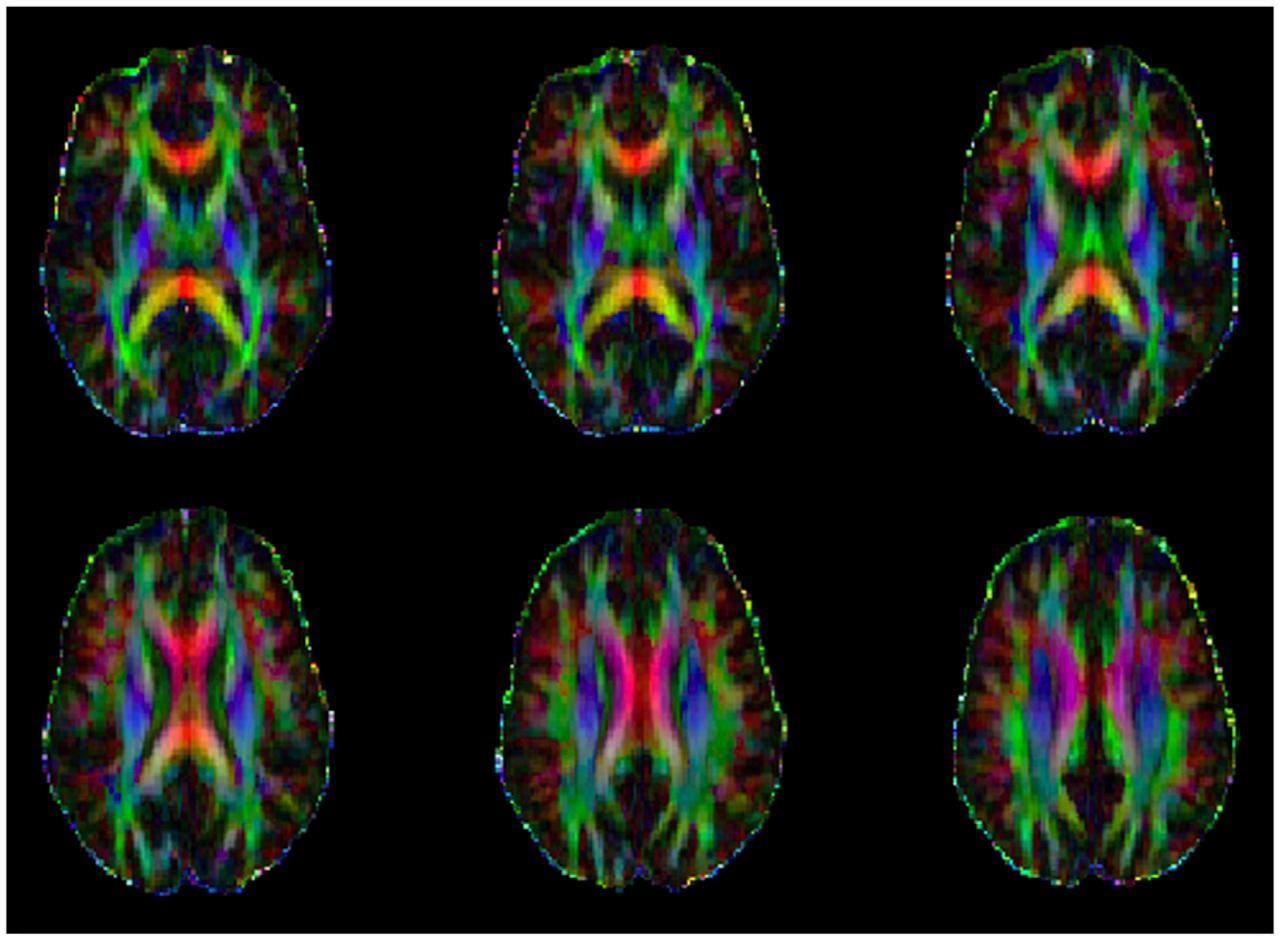
**Example of a series of color orientation fractional anisotropy maps from one study participant where the primary colors represent principal directions of the various white-matter tracks (red: left–right, green: anterior–posterior, and blue: superior–inferior)**.

To examine the relationship between WM integrity and blast injury, we correlated the total number of blast exposures with DTI FA values across all participants. Increased blast exposure was associated with decreased FA in several WM tracts, including the corpus callosum, the corticospinal tract, the internal capsule, and the cingulum (Figure 2; Table 5). When cingulum was added as a covariate, the relationship between blasts and overall FA remained significant for the right (SE 0.0003; *p* = 0.04), but not for the left, cingulum (SE 0.0003; *p* = 0.32). This is in line with other reports suggesting that cingulum is a vulnerable area, which is often affected in blast injury (26, 51, 52) and may account for a significant portion of the findings. However, the overall FA measures survived significance for the right cingulum suggesting that the relationship between number of blasts and abnormalities in brain microstructure extends beyond that particular brain region. Additionally, the ANCOVA analyses indicated that there were no between-group differences in FA based on mTBI diagnosis (i.e., main effect for mTBI controlling for age and education was *p* > 0.74).

**Table 5:** **White matter integrity and total blast exposure**.

Tract	*n*	*r*	*p*
Splenium (corpus callosum)	40	−0.34	0.033
Corticospinal tract R	40	−0.47	0.002
Corticospinal tract L	40	−0.39	0.014
Retrolenticular internal capsule R	40	−0.32	0.041
Posterior corona radiata R	40	−0.39	0.013
Sagittal stratum L	40	−0.35	0.028
Cingulum bundle R	40	−0.31	0.057
Cingulum bundle L	40	−0.46	0.009
Fornix striata terminalis L	40	−0.40	0.012
Uncinate fasciculus L	40	−0.33	0.041
Tapetum R	40	−0.53	0.001
Tapetum L	40	−0.32	0.043

*R, right; L, left*.

### Predicting FA Values

Multiple linear regression analysis was employed to develop models predicting WM integrity. A first set of models predicted composite FA (i.e., averaged FA across 48 WM tracts) from PTSD symptom severity, total blast exposure, LOC, the four cognitive scores (memory domain score, language/education domain score, attention/executive function domain score, and overall composite score), age, and education. A model was developed including each cognitive score singly, resulting in four separate models of FA composite. Our results show that blast exposure predicts composite FA in the model including the attention/executive function domain score, such that as blast exposure increases, FA decreases (Table 6). Specifically, blast exposure explains 15% of the variability in composite FA scores when including the attention/executive function domain score. The six-predictor model was statistically significant, *F*_(6,25)_ = 3.42, *p* = 0.01, and accounted for 45% of the variance in composite FA (*R*^2^ = 0.45, adjusted *R*^2^ = 0.32).

**Table 6:** **Predicting composite fractional anisotropy (FA) scores**.

Outcome	Predictor	Slope	SE	*p*-Value	*R*^2^	Adjusted *R*^2^	Semi-partial *R*^2^	Partial *R*^2^
FA composite	PTSD	−0.00023	0.00020	0.2723	0.4508	0.3190	0.02768	0.04798
	# BLASTS	−0.00082	0.00031	**0.0137**			0.15460	0.21967
	Age	−0.00066	0.00051	0.2151			0.03555	0.06079
	Education	0.00256	0.00175	0.1561			0.04697	0.07879
	LOC1n2y	−0.00574	0.00700	0.4203			0.01475	0.02616
	**Attention**	0.02010	0.05147	0.6994			0.00335	0.00606
	PTSD	−0.00025	0.00019	0.2138	0.4728	0.3462	0.03433	0.06113
	# BLASTS	−0.00073	0.00031	**0.0281**			0.11464	0.17860
	Age	−0.00086	0.00046	0.0734			0.07364	0.12255
	Education	0.00377	0.00174	**0.0401**			0.09892	0.15797
	LOC1n2y	−0.00588	0.00686	0.3996			0.01549	0.02853
	**Language**	−0.03536	0.03228	0.2838			0.02531	0.04580

*PTSD, post-traumatic stress disorder; LOC, loss of consciousness*.

*p-values in red are significant at p < 0.05 level*.

**Figure 3:**
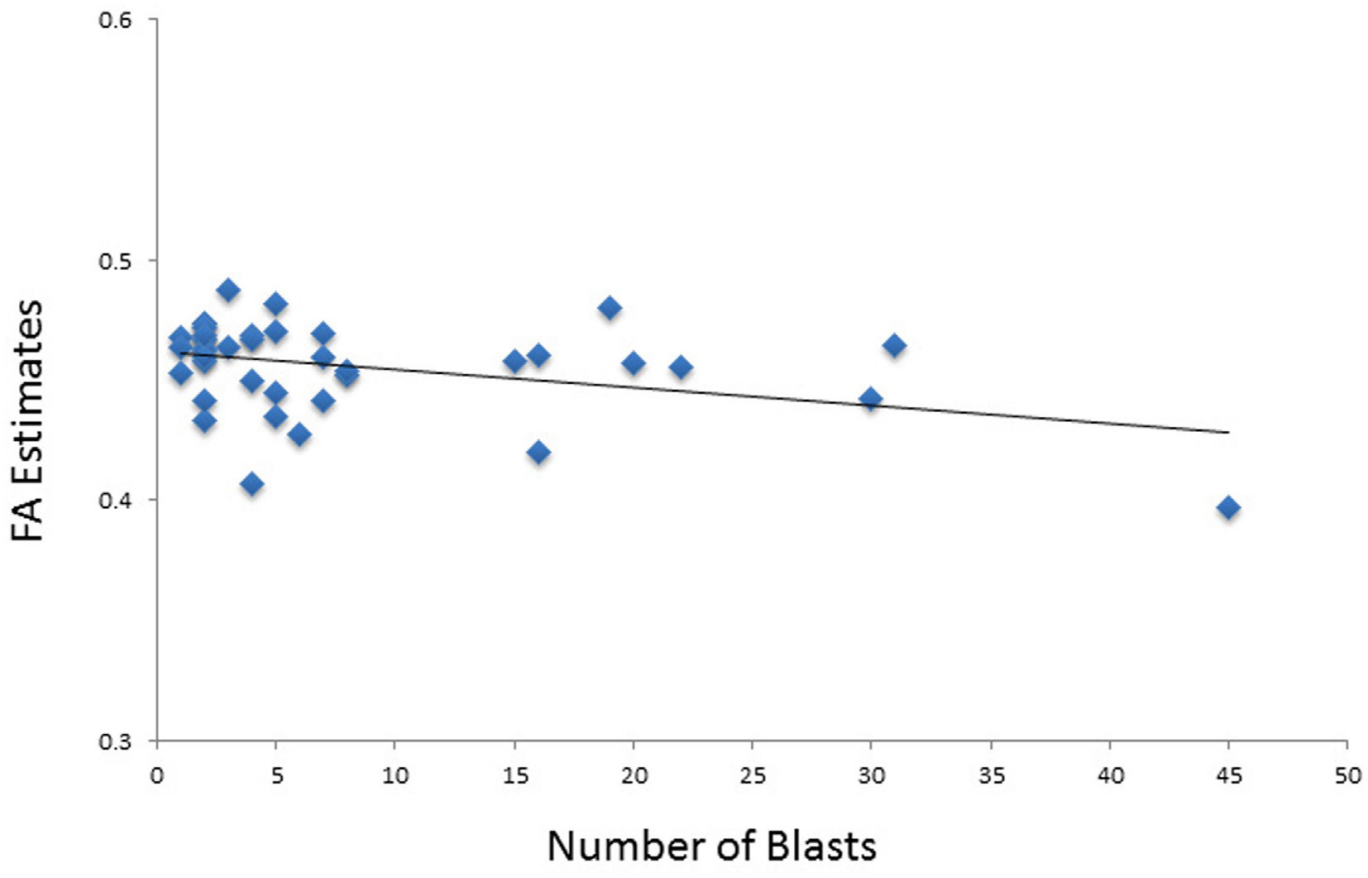
**Scatterplot shows the relationship between total number of blasts and fractional anisotropy (FA) values**.

In the model of composite FA including the language domain scores, both total blast exposure and education had significant (*p* < 0.05) partial effects (Table 6). Total blast exposure explained 11% of the variability in this model, predicting composite FA such that as blast exposure increases, FA decreases (Figure 3). Education explained 10% of the variability in this model, predicting FA composite such that as the level of education increases, FA increases. The six-predictor model was statistically significant *F*_(6,25)_ = 3.74, *p* < 0.01, and accounted for 47% of the variance in composite FA (*R*^2^ = 0.47, adjusted *R*^2^ = 0.35).

We also examined FA individually in the right and left cingulum bundle. The independent variables included in these models were PTSD symptom severity, total blast exposure, LOC, the four cognitive scores, age, and education. Blast exposure and education had significant (*p* < 0.05) partial effects in two of the four models examining FA in the right cingulum bundle (Table 7). Our results indicate that education predicts FA in the right cingulum bundle such that as the level of education increases, FA increases. Education accounts for the largest share of the variability in FA in the right cingulum (25% of the model with neurocognitive composite scores and 32% with memory scores). Total blast exposure also significantly predicts FA in the right cingulum bundle, such that as blast exposure increases, FA decreases. The total number of blasts accounts for 18% of the variability of the model with the neurocognitive composite score and 19% of the variability of the model with the memory domain score.

**Table 7:** **Predicting fractional anisotropy in the right cingulum**.

Outcome	Predictor	Slope	SE	*p*-Value	*R*^2^	Adjusted *R*^2^	Semi-partial *R*^2^	Partial *R*^2^
R cingulum FA	Post-traumatic stress disorder (PTSD)	0.00001	0.00026	0.9602	0.5061	0.3826	0.00005	0.00011
	# BLASTS	−0.00155	0.00052	**0.0072**			0.17743	0.26429
	Age	−0.00093	0.00062	0.1445			0.04684	0.08661
	Education	0.00704	0.00203	**0.0020**			0.24718	0.33353
	Loc1n2y	−0.00525	0.00868	0.5508			0.00753	0.01502
	**Composite**	0.04110	0.04880	0.4079			0.01460	0.02871
	PTSD	0.00006	0.00026	0.8199	0.5231	0.4038	0.00105	0.00220
	# BLASTS	−0.00158	0.00051	**0.0049**			0.19048	0.28539
	Age	−0.00090	0.00060	0.1432			0.04552	0.08713
	Education	0.00768	0.00192	**0.0005**			0.31691	0.39921
	Loc1n2y	−0.00494	0.00854	0.5680			0.00666	0.01378
	**Memory**	0.03694	0.02930	0.2195			0.03159	0.06211

*p-values in red are significant at p < 0.05 level*.

Total blast exposure, age, and education were significant predictors of FA in the left cingulum bundle (Table 8). Blast exposure predicts FA in the left cingulum such that as the number of blasts increases, FA decreases. Specifically, blast exposure accounts for between 12 and 18% of variability in FA in the left cingulum. Age also inversely predicts FA in the left cingulum bundle, accounting for approximately 11% of variability in FA. Education accounted for the largest share of variability in FA in the left cingulum (23–27%) and predicts FA such that as the level of education increases, FA increases. When other individual WM tracts (listed in Table 5) were examined, nothing was found to be significant.

**Table 8:** **Predicting fractional anisotropy in the left cingulum**.

Outcome	Predictor	Slope	SE	*p*-value	*R*^2^	Adjusted *R*^2^	Semi-partial *R*^2^	Partial *R*^2^
L cingulum FA	Post-traumatic stress disorder (PTSD)	0.00009	0.00024	0.7184	0.4943	0.3679	0.00281	0.00552
	# BLASTS	−0.00122	0.00048	**0.0183**			0.13517	0.21091
	Age	−0.00131	0.00056	**0.0286**			0.11429	0.18434
	Education	0.00639	0.00185	**0.0020**			0.25208	0.33266
	LOC1n2y	−0.01095	0.00789	0.1778			0.04060	0.07433
	**Composite**	−0.03436	0.04436	0.4461			0.01264	0.02439
	PTSD	0.00014	0.00025	0.5693	0.4854	0.3567	0.00714	0.01368
	# BLASTS	−0.00138	0.00048	**0.0079**			0.18025	0.25939
	Age	−0.00111	0.00056	0.0569			0.08584	0.14295
	Education	0.00605	0.00180	**0.0026**			0.24316	0.32087
	LOC1n2y	−0.01051	0.00797	0.1994			0.03735	0.06767
	**Memory**	0.01135	0.02734	0.6819			0.00369	0.00712
	PTSD	0.00015	0.00022	0.4878	0.5635	0.4587	0.00866	0.01945
	# BLASTS	−0.00104	0.00034	**0.0050**			0.16564	0.27506
	Age	−0.00142	0.00056	**0.0183**			0.11129	0.20315
	Education	0.00699	0.00191	**0.0012**			0.23449	0.34945
	LOC1n2y	−0.01104	0.00764	0.1607			0.03648	0.07713
	**Attention**	−0.07773	0.05615	0.1785			0.03346	0.07119
	PTSD	0.00012	0.00021	0.5915	0.5977	0.5011	0.00476	0.01168
	# BLASTS	−0.00092	0.00033	**0.0102**			0.12428	0.23601
	Age	−0.00129	0.00049	**0.0149**			0.11003	0.21476
	Education	0.00763	0.00186	**0.0004**			0.27000	0.40160
	LOC1n2y	−0.01070	0.00733	0.1569			0.03427	0.07850
	**Language**	−0.07077	0.03451	0.0509			0.06769	0.14403

*p-values in red are significant at p < 0.05 level*.

### Predicting Cognitive Functioning

Multiple linear regression analysis was employed to develop models predicting cognitive functioning. Each of the four cognitive scores was examined as a dependent variable. PTSD symptom severity, total blast exposure, LOC, and FA were the primary independent variables while adjusting for age and education. We found no significant partial effects of these primary independent variables in multiple regression models predicting neurocognition.

## Discussion

We examined the relationship between blast exposure and WM integrity using DTI and neurocognitive assessments in a cohort of Iraq and Afghanistan combat veterans who had multiple exposures during deployment but no formally diagnosed history of head trauma before military service. Two sets of findings emerged: first, reduced FA was associated with greater blast exposure, but not with the clinical diagnosis of mTBI. Second, while the blast-exposed veterans scored below the expected performance for their age on tasks of memory and language, participants with a clinical diagnosis of mTBI were more impaired than their blast-exposed non-TBI counterparts on these tasks, as well as on tasks of attention and executive functioning.

### WM Integrity

Consistent with the hypotheses, the results suggest that WM integrity is compromised in veterans with blast exposure in relation to the dose of exposure, which corroborates the findings of a recent study by Trotter et al. (53). However, our findings are the first to show this dose–response relationship regardless of whether exposures were severe enough to result in LOC and/or clinical mTBI diagnosis. When examining FA in individual WM tracts, we found that greater blast exposure significantly predicted lower FA in the bilateral cingulum. The cingulum bundle is a complex structure that has been implicated in memory formation and executive function (54) as it contains prominent medial and dorsal prefrontal connections to the medial temporal and parietal lobes (55). It is uniquely vulnerable to cerebral trauma given its shape and location within the skull (56), and lasting changes to WM in this region following moderate–severe civilian mTBI have been reported (26, 57).

A study by Wu et al. (58) examined 12 civilian mTBI participants and 10 matched non-TBI controls. They reported that decreased FA in the bilateral cingulum bundle was associated with lower performance on an episodic verbal learning and memory task in the mTBI group, while no association was observed in controls (58). We did not find significant correlations between neuropsychological performance and FA, and cognitive domain scores were not significant predictors of WM changes either in models examining composite or individual WM tract FA. This suggests that the biological mechanisms that underlie cognitive deficits in the acute stages of mTBI (58) may differ from those still present years after deployment (our sample averaged 3.7 ± 1.9 years post-deployment). In clinical terms, this suggests that there might be a natural trajectory for the traumatized brain to recover and compensate for the effects of acute blast-related trauma as suggested by others (30). One can further hypothesize that early initiation of rehabilitation cognitive programs is warranted in veterans with history of blast exposures even if they may not meet criteria for TBI.

Blast exposure is thought to inflict injury through pressure waves transmitted in the air. Damage to the nervous system is thought to occur through biophysical mechanisms related to the shock wave’s impact on brain tissue (59–61). Blast injuries severe enough to cause moderate-to-severe TBI are without doubt a mix of injury mechanisms common to both blast and non-blast forms of injury. What is not well understood is the degree to which primary blast waves, particularly those of lesser intensity, may injure the brain directly, although studies in experimental animal models suggest that even relatively low-level blast exposure can cause direct injury (18, 62–64). This last notion potentially explains our finding that greater number of blast exposures is associated with compromised WM integrity or decreased FA, even in the absence of the clinical symptoms of mTBI. Nonetheless, FA changes in the cingulum in individuals with multiple blast exposures may represent a marker of vulnerability for future cognitive deficits.

### WM Integrity and Education

Differences in WM integrity between individuals with varying levels of education may have influenced and offset the FA changes related to blast exposure. Education significantly predicted FA in both the left and the right cingulum, in the opposite direction as blast exposure. Education is thought to play a role in developing the WM microstructure and several groups have linked higher levels of education to higher FA (65, 66). Noble et al. (66) found that higher education is an age-independent predictor of WM integrity in late adolescence, and that greater educational attainment was associated with increased FA in the superior longitudinal fasciculus and in the cingulum bundle. Together, these observations suggest that higher levels of education before exposure to blast(s) could serve as a resilience factor that may help to mitigate the negative effects of blast injury on cognition. Moreover, a veteran’s education level may serve as an additional guide in developing rehabilitation programs that optimize clinical outcomes.

### mTBI and PTSD

Post-traumatic stress disorder in veterans has been shown to negatively affect cognition (67, 68) and the contribution of individual disorders (e.g., TBI vs. PTSD) to cognitive deficits in veterans exposed to blast and other injuries remains a subject of debate. Our findings indicate that veterans who met criteria for mTBI indeed performed worse on a battery of neurocognitive tests in comparison to those who did not meet criteria for the clinical diagnosis of mTBI; also, we did not find a significant effect of PTSD on neuropsychological testing performance. As this study lacked a PTSD-only control group, this finding should be interpreted with caution. In clinical terms, if assumed that mTBI may account for most of the cognitive deficits in cases of comorbid mTBI and PTSD, then it is expected that improvements in cognition should be associated with TBI-focused treatments. However, as all diagnosed conditions deserve the appropriate treatment to be delivered simultaneously, the initiation of cognitive rehabilitation regardless of diagnoses seems prudent when cognitive difficulties are suspected or identified.

### WM Integrity and Cognitive Functioning

Prior studies linking mTBI to decreased FA have suggested, by extension, that a loss of WM integrity could underlie cognitive deficits (18, 34, 53, 69–72). However, our hypothesis that lower FA would be associated with poorer performance on neuropsychological tests across cognitive domains was not supported. WM abnormalities did not explain the neurocognitive deficits. It is interesting in this context to note that only a minority of participants without an mTBI diagnosis (4 of the 16 veterans) had secondary or tertiary injuries, suggesting that most of these participants experienced only the effects of primary blast. By contrast, most veterans with a mTBI diagnosis experienced secondary or tertiary injuries suggesting a more mixed blast/non-blast mechanism of injury. Thus, while primary blast may alter WM integrity, the production of significant cognitive defects may require a combination of blast with non-blast secondary and tertiary injuries or a higher intensity blast exposure. This suggests considerable heterogeneity in relation to the causes for mTBI, and it is also possible that the cognitive deficits observed in veterans with mTBI were subserved by mechanisms other than changes in WM integrity. What is still unknown is the extent to which the cumulative effects of multiple blasts on WM integrity may confer vulnerability for neurological or psychological conditions and lead to the early development of neurodegenerative pathology. Further work is needed in order to elucidate the mechanism by which blast exposure affects brain structure in order to be able to develop potential therapeutics.

### Study Limitations and Future Research

As is typical of neuroimaging research, there are limitations to the present study. The imaging protocol was conducted in accordance with the well-accepted and verified image acquisition techniques available at the time of the study. We acknowledge that there have been more recent improvements in the methodology of DTI image acquisition that offer certain advantages over the protocol used in this study (e.g., no gap in image acquisition). It should be emphasized, however, that all measures were taken to assure the highest possible quality of the neuroimaging data at the time of the study. Some investigators have suggested that improvements in data quality and implementation of more sophisticated tractography methods are unlikely to lead to increasingly accurate maps of human anatomical connections (73). Additionally, it is possible that the use of more specialized neuropsychological tests [or subsections of such tests, see Ref. (12, 13)] may have identified and isolated more discrete cognitive deficits. Such detailed assessments may have provided measures of cognitive impairment that might have been more associated with the biological measures of WM integrity. On the other hand, such assessments would have also increased the burden to participants. The total number of participants was limited to 40 making additional work important for replication of the study’s findings, especially with the inclusion of an additional control group with no blast exposure. Finally, the inclusion of a PTSD-only group would have helped to delineate WM profiles uniquely linked to PTSD; however, this was not a primary aim of the study. Future research is needed to address these issues.

## Conclusion

This report documents new evidence that a greater total number of blast exposures is associated with lower WM integrity even in the absence of clinical TBI. Further, as the etiology of blast-related TBI is rather diverse, specific FA changes in the cingulum in veterans with multiple blast exposures is hypothesized to be a marker of vulnerability for future cognitive deficits.

## Author Contributions

EMM was the PI of a VA Merit Grant that supported this research. CF drafted some sections of the paper and assisted with the data collection. II and DD wrote sections of the paper. EW and CT assisted in image processing and the statistical analysis of the neuroimaging data. JS and CB helped recruit and assess the study participants. EM consulted on the statistical analysis. GE worked on the paper and consulted on the research study. MS consulted on all aspects of the neuropsychology data. EH was a co-investigator on the VA Merit Grant, worked on the statistical analysis, data interpretation, and the paper.

## Disclaimer

The opinions expressed herein are those of the authors and are not necessarily representative of those of the Uniformed Services University of the Health Sciences, the Department of Defense; or, the United States Army, Navy, or Air Force (DLD).

## Conflict of Interest Statement

The authors declare that the research was conducted in the absence of any commercial or financial relationships that could be construed as a potential conflict of interest.

## Footnotes

^1^http://www.ptsd.va.gov/professional/pages/assessments/assessment-pdf/PCL-handout.pdf.

^2^http://www.fmrib.ox.ac.uk/fsl/.

## Abbreviations

COWA-FAS, Controlled Oral Word Association Test-FAS; CVLT, California Verbal Learning Test; DoD, Department of Defense; DTI, diffusion tensor imaging; DVBIC, Defense and Veterans Brain Injury Center; FA, fractional anisotropy; FOV, field of view; IED, improvised explosive device; ISMMS, Icahn School of Medicine at Mount Sinai; JJPVAMC, James J. Peters Veterans Affairs Medical Center; MD, mean diffusivity; MRI, magnetic resonance imaging; mTBI, mild traumatic brain injury; PCL-M, PTSD Checklist-Military Version; PTSD, post-traumatic stress disorder; TBI, traumatic brain injury; TBSS, tract-based spatial statistics; TE, echo time; TR, repetition time; VA, Veteran’s Affairs; WM, white matter.
